# N‐Doped Porous Carbon Based on Anion and Cation Storage Chemistry for High‐Energy and Power‐Density Zinc Ion Capacitor

**DOI:** 10.1002/advs.202407635

**Published:** 2024-10-07

**Authors:** Yuanyuan Liang, Miaomiao Wu, Anjie Liu, Qihua Chen, Yan Wu, Qian Xiang, Zhibo Liu, Jixi Guo, Xingchao Wang, Dianzeng Jia

**Affiliations:** ^1^ State Key Laboratory of Chemistry and Utilization of Carbon Based Energy Resources Insitute of Applied Chemistry, College of Chemistry Xinjiang University Urumqi Xinjiang 830046 P. R. China; ^2^ Key Laboratory of Advanced Energy Materials Chemistry (Ministry of Education) Nankai University Tianjin 300071 P. R. China

**Keywords:** anion and cation storage mechanism, coal pitch, hybrid zinc‐ion capacitor, N‐doped porous carbon, quasi‐solid‐state

## Abstract

Zinc ion hybrid capacitors (ZIHCs) show promise for large‐scale energy storage because of their low cost, highly intrinsic safety, and eco‐friendliness. However, their energy density has been limited by the lack of advanced cathodes. Herein, a high‐capacity cathode material named N‐doped porous carbon (CFeN‐2) is introduced for ZIHCs. CFeN‐2, synthesized through the annealing of coal pitch with FeCl_3_·6H_2_O as a catalytic activator and melamine as a nitrogen source, exhibits significant N content (10.95 wt%), a large surface area (1037.66 m^2^ g^−1^), abundant lattice defects and ultrahigh microporosity. These characteristics, validated through theoretical simulations and experimental tests, enable a dual‐ion energy storage mechanism involving Zn^2+^ ions and CF_3_SO_3_^−^ anions for CFeN‐2. When used as a cathode in ZIHCs, CFeN‐2 achieves a high‐energy density of 142.5 W h kg^−1^ and a high‐power density of 9500.1 W kg^−1^. Furthermore, using CFeN‐2 ZIHCs demonstrate exceptional performance with 77% capacity retention and nearly 100% coulombic efficiency after 10 000 cycles at 10 A g^−1^, showcasing substantially superior performance to current ZIHCs. This study offers a pathway for developing high‐energy and high‐power cathodes derived from coal pitch carbon for ZIHC applications.

## Graphical abstract

The synthesized CFeN‐2 based on significant nitrogen content (10.95 wt%), large specific surface area (1037.66 m^2^g^−1^), and a dual‐ion (Zn^2+^ and CF_3_SO_3_^−^) energy storage mechanism, demonstrates a high energy density of 142.5 Wh kg^−1^, a high power density of 9500.1 W kg^−1^, and remarkable cycling stability (CE ≈100% after 10 000 cycles at 10 A g^−1^).

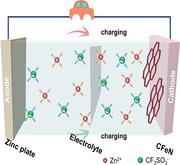

## 1 Introduction

Lithium‐ion batteries are widely used in portable electronics and electric vehicles due to their high energy density and excellent cycle stability, but the safety risks of toxic and flammable organic electrolytes remain a challenge.^[^ ^1^, ^2^, ^3^, ^4^, ^5^ ^]^ In recent years, aqueous‐based rechargeable batteries have emerged as a promising alternative due to their cost‐effectiveness, safety, eco‐friendliness, and high ionic conductivity^[^ ^6^, ^7^, ^8^, ^9^ ^]^ Among these, aqueous zinc‐ion hybrid capacitors (ZIHCs) have shown potential as the next generation of energy storage devices by combining the advantages of battery‐type Zn anodes and capacitor‐type cathodes.^[^ ^4^, ^10^, ^11^ ^]^ Zn metal anodes offer attractive merits such as a low redox potential (0.76 V vs SHE), high theoretical capacity (820 mA h g^−1^), and large specific volumetric capacity (5855 mA h cm^−3^), along with a fast plating/stripping process.^[^ ^12^, ^13^, ^14^, ^15^ ^]^ Carbon‐based electrodes in the cathodes enable high power density through an ultrafast adsorption/desorption mechanism.^[^ ^16^, ^17^, ^18^, ^19^, ^20^, ^21^, ^22^, ^23^, ^24^, ^25^, ^26^ ^]^ Furthermore, the natural abundance, considerably low cost, non‐toxicity, and intrinsic safety of Zn anodes and carbon‐based cathodes, along with aqueous electrolytes, make ZIHCs more suitable for large‐scale energy storage applications.

Inspired by these advantages, an increasing number of research efforts and research groups have shifted toward developing ZIHCs in recent years. For instance, Wei et al prepared N, S blended porous carbon (NSPC) as the cathode material for ZIHCs by using biomass as a raw material.^[^ ^27^ ^]^ A considerable capacity of 86.5 mA h g^−1^, with an energy density of 122.6 W h kg^−1^ for ZIHCs was obtained at the current density of 20 A g^−1^. Liu's group used chitosan as a raw material to produce nitrogen‐doped graded porous carbon, delivering an energy density of 92.5 W h kg^−1^ at a power density of 3633.4 W kg^−1^ in ZIHCs.^[^ ^28^ ^]^ Additionally, 2D layered B/N co‐doped porous carbon, derived from acrylonitrile copolymer, exhibited a high energy density of 86.8 W h kg^−1^ and power density of 12.2 kW kg^−1^ within a voltage window of 0.2–1.8 V.^[^ ^29^ ^]^ Despite these advancements, the energy density and long‐term cycle performance of ZIHCs still lag far behind the growing requirements, especially at high current densities, posing a great challenge for broader large‐scale applications. Consequently, the development of ZIHCs with superior energy density, high power density, and extended cycle life remains an ongoing challenge.

In this work, we construct a robust ZIHCs with high energy density and power density by employing coal‐based N‐doped porous carbon (CFeN‐2) as a new promising cathode and Zn metal as the anode in an aqueous electrolyte. CFeN‐2, characterized by a high N‐doping (atomic mass percentage of 10.95 wt%) level and poriferous architecture, was obtained through a simple calcination process of coal pitch with ferric chloride as a catalyst and melamine as a nitrogen source. The unique architecture of CFeN‐2 offers several advantages in addressing the shortcomings of carbon cathodes in ZIHCs. i) the coexistence of micropores and mesopores in CFeN‐2 simultaneously provide sufficient zinc storage sites and rapid ion transport pathways. ii) the unique structure effectively tunes favorable surface chemistry for improved electrolyte wettability. iii) nitrogen atoms exhibit strong binding with Zn^2+^ ions, offering additional active sites. These advantages allow the CFeN‐2 material to provide a high capacity of 150 mAh g^−1^ and an energy density of 142.5 Wh kg^−1^ at a current density of 1 A g^−1^, representing one of the best performance among carbon‐based ZIHCs. Furthermore, using CFeN‐2 demonstrates a prominent energy density of 95.1 W h kg^−1^ at a power density of 9500.1 W kg^−1^. More importantly, a pouch‐type quasi‐solid‐state ZIHCs using CFeN‐2 can power the timepiece for up to 10 days or more.

## 2 Results and Discussion

**Scheme 1:**
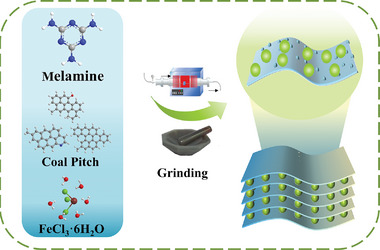
Schematic illustration of synthesis process for CFeN.

**Figure 1:**
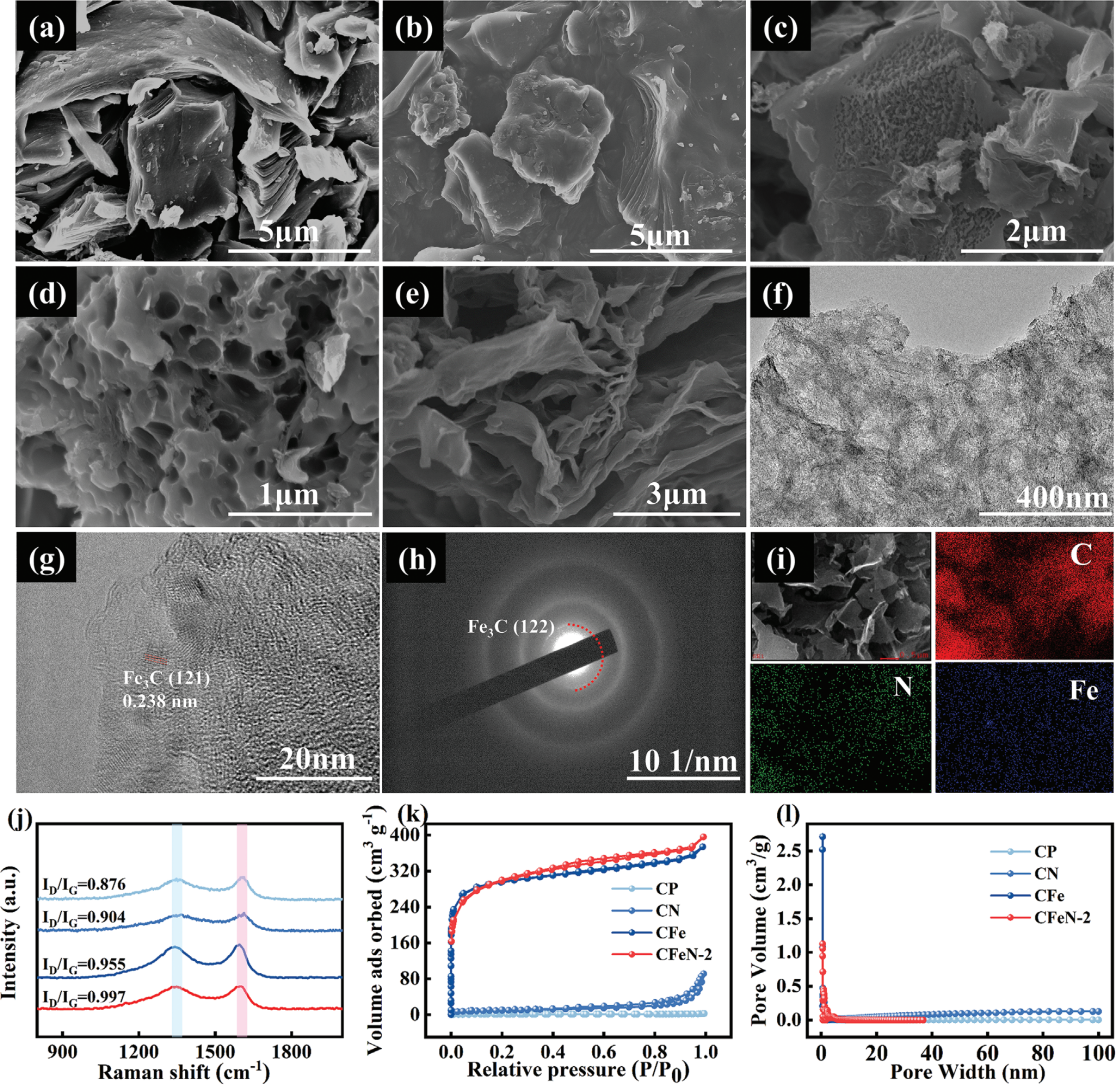
Structural Characterizations. SEM images of a) raw coal pitch, b) CP, c) CN, d) CFe; e) SEM, f,g) HRTEM, h) SAED pattern, and i) mapping images of CFeN‐2. j) Raman spectra, k) N_2_ adsorption–desorption curve, l) pore size distribution of CP, CN, CFe, and CFeN‐2.

**Scheme** **1** depicts the fabrication of the hierarchical CFeN architecture using coal pitch as the carbon source, melamine as the nitrogen dopant and FeCl_3_·6H_2_O as the activation agent. These ingredients were first mixed together by grinding and then carbonized at 800 °C, a temperature chosen based on the results of thermogravimetric analysis (Figure S1, Supporting Information). To remove any residual iron catalyst, the material was subsequently washed with a hydrochloric acid solution, yielding the production of N‐doped porous carbon (CFeN). The morphologies of raw coal pitch, heat‐treated coal pitch (CP), CN, CFe, and CFeN samples were investigated using scanning electron microscopy (SEM) (**Figure** **1**a–e). It was observed that raw CP displayed irregular bulk particles and layered bulk structures. CP and CN samples exhibited similar morphologies, but with a carbon layer wrapping around the surface due to the thermal decomposition of small molecules in the raw material. In contrast, the CFe sample showcased a rich hollow pore structure, attributed to the assistance of FeCl_3_·6H_2_O as a catalyst and pore‐forming agent. Furthermore, CFeN sample also revealed a distinctive lamellar hierarchical porous nanosheet structure due to the presence of melamine as a nitrogen dopant and FeCl_3_·6H_2_O as a catalyst.

To further investigate the impact of melamine content on sample morphology, three samples with different melamine contents were used for the study (Figure S2, Supporting Information). At the melamine mass ratio of 1:11:2 (coal pitch: melamine: FeCl_3_·6H_2_O) (CFeN‐2), the CFeN‐2 sample maintained a consistent lamellar structure similar to CFeN‐1 (1:11:1), but with a more orderly arrangement. However, as the melamine mass ratio increased to 1:11:3 (CFeN‐3), the sample displayed some broken nanoparticle‐formed agglomerates, resulting from the high melamine content and strong van der Waals attraction between carbon layers. Therefore, CFeN‐2 sample was identified as the optimal sample for further investigation.

The laminar‐graded porous nanosheet structure of CFeN‐2 was further confirmed by transmission electron microscopy (TEM). The TEM images of CFeN‐2 displayed thin wrinkles and excellent dispersion, presenting a network of interconnected sheet‐like structures (Figure 1f; Figure S3, Supporting Information). In addition, the HRTEM image and SAED pattern of CFeN‐2 confirmed the presence of Fe_3_C (Figure 1g,h). Energy dispersive X‐ray spectroscopy (EDS) was employed to investigate the elemental composition of CFeN‐2, ultimately proving the uniform distribution of C, N, and Fe elements on its surface, thus demonstrating successful N doping and Fe_3_C formation (Figure 1i; Figure S4, Supporting Information). Moreover, the atomic mass percentage of N in CFeN‐2 was measured at 10.95 wt%, significantly higher than that of CP (2.36 wt%) (Table S1, Supporting Information). Elevating N doping in carbon materials is crucial in regulating structural organization, enhancing conductivity, facilitating electrolyte penetration, and creating additional Zn^2+^ active sites through chemisorption.

X‐ray diffraction (XRD) patterns were utilized to analyze the physical phase composition of CP, CN, CFe, and CFeN‐2 (Figure S5, Supporting Information). The peaks at ≈25° and ≈43°, corresponded to the (002) and (100) crystal planes of graphite carbon in these materials. Interestingly, the intensity of the diffraction peaks on the crystal plane of CFeN‐2 (002) was significantly higher than that of the other samples, indicating a higher graphite crystallinity and enhanced electronic conductivity of CFeN‐2. Notably, the XRD pattern of CFe activated with FeCl_3_ exhibited characteristic peaks of Fe_2.9_O_4_ (PDF#86‐1348), while the XRD pattern of CFeN‐2 revealed a characteristic peak of Fe_3_C (PDF#35‐0772), indicating a complete conversion of Fe_2.9_O_4_ to Fe_3_C post the addition of melamine carbonation.^[^ ^30^, ^31^ ^]^ These findings imply that the conversion of iron compounds during carbonization follows the specific Equations ((1), (2), (3), (4)). The amorphous iron compound (Fe(OH)_3_, FeO(OH)) underwent a two‐step conversion process. Initially, it was converted to Fe_2_O_3_ at 673 °C (Equation 4). Subsequently, at temperatures exceeding 973 °C, it was further reduced to Fe_3_O_4_. Upon additional reduction with amorphous carbon, it is transformed into Fe_3_C and Fe metal. Furthermore, the XRD patterns of CFeN‐1, CFeN‐3, and CFeN‐4, each with varying melamine mass, exhibited significant Fe_3_C (PDF#35‐0772) index diffraction peaks (Figure S6, Supporting Information).

(1) $$\mathrm{FeC}l_{3}\cdot 6H_{2}O\to \mathrm{Fe}{\left(\mathrm{OH}\right)}_{3}\to \mathrm{FeO}\left(\mathrm{OH}\right)\to Fe_{2}O_{3}$$

(2) $$Fe_{2}O_{3}+\left(H_{2},\;C,\;\mathrm{CO}\right)\to Fe_{3}O_{4}$$

(3) $$Fe_{3}O_{4}+\left(H_{2},\;C,\;\mathrm{CO}\right)\to \mathrm{Fe}$$

(4) $$\mathrm{FeO}+C\to Fe_{3}C$$

Raman spectra of the samples exhibited characteristic peaks (D and G peaks) at 1350 and 1580 cm^−1^, respectively, which are characteristics of carbon structure (Figure 1j).^[^ ^32^ ^]^ The *I*_D_ */I*_G_ values of CFe (0.955) and CN (0.904) were higher than that of CP (0.876), indicating that the presence of activators and N heteroatoms reduced the degree of graphitization on the carbon matrix material surface. Interestingly, the *I_D_/I_G_* value of CFeN‐2 (0.997) increased with the simultaneous introduction of FeCl_3_·6H_2_O and melamine, demonstrating a synergistic effect between the two on the material's structure.

The specific surface area (SSA) and pore size distribution (PSD) of CP, CN, CFe, and CFeN‐2 samples were analyzed using N_2_ adsorption/desorption isotherms. All samples exhibited IV‐typed isotherms with noticeable absorption at P/P_0_ < 0.1 and a lagging hysteresis at 0.2 < P/P_0_ < 1, verifying their mesoporous characteristics. The pore properties of these samples are summarized in Table S2 (Supporting Information). Notably, the specific Brunauer‐Emmett‐Teller surface area (S_BET_) of CFe (1182.45 m^2^ g^−1^) was considerably higher than that of CN (32.91 m^2^ g^−1^) and CP (2.02 m^2^ g^−1^), emphasizing the effect of FeCl_3_ as an activator in creating multiple pore structures in the carbon matrix (Figure 1k). Compared with CFe (1182.45 m^2^ g^−1^), CFeN‐2 exhibits a reduced specific surface area of 1037.66 m^2^ g^−1^, a consequence of nitrogen of N‐atom doping that alters the microstructure of the porous carbon and increases its active sites. Micropores can enhance the material surface area, while mesopores can improve the ion transport of electrolyte and decrease ion transport resistance.^[^ ^33^ ^]^ The PSD analysis of CFeN‐2 confirmed its hierarchical porosity, predominantly composed of micropores (0.55 nm) and mesopores (2.20 nm) (Figure 1l). CFeN‐2 demonstrated a higher proportion of mesopores (73.76%) compared to CFe‐2 (71.90%), facilitating rapid electrolyte infiltration, enhancing charge transfer rate, and providing adequate active sites for boosting capacity and energy density. Therefore, it can be concluded that CFeN‐2 features an appreciable specific surface area and a micro‐mesoporous structure, allowing it to exhibit excellent electrochemical performances required in various applications.

**Figure 2:**
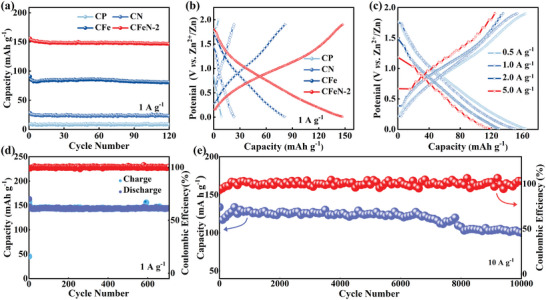
Electrochemical performance. a) Cycling performance of CP, CN, CFe, and CFeN‐2 cathodes at 1 A g^−1^. b) GCD curves of CP, CN, CFe, and CFeN‐2 cathodes at 1 A g^−1^. c) Charge‐discharge curves of CFeN‐2 cathode at current densities from 0.5 to 5 A g^−1^. Long cycling performance of CFeN‐2 cathode at d) 1 A g^−1^ and e) 10 A g^−1^, respectively.

The electrochemical performance of the samples as electrode materials for ZIHCs was evaluated. Cycling performance and galvanostatic charge/discharge (GCD) curves at 1 A g^−1^ confirmed that CFeN‐2 cathode exhibited the highest specific capacity (150 mAh g^−1^) compared to CP (8 mAh g^−1^), CN (25 mAh g^−1^) and CFe (85 mAh g^−1^) cathodes (**Figure** **2**a,b). In addition, the electrochemical performance of the samples with varying FeCl_3_·6H_2_O additions was further compared to investigate the effect of component contents on CFeN‐2, highlighting the exceptional specific capacity (82 mAh g^−1^) of CFe‐2 (Figure S7, Supporting Information). These findings provide additional support for the role of FeCl_3_·6H_2_O in creating appropriate specific surfaces, pore structures, and active sites for materials. The impact of melamine on the electrochemical performance of CFeN‐X (X = 1, 2, 3) was also investigated, with CFeN‐2 exhibiting the highest discharge capacity (Figure S8, Supporting Information), indicating promising electrochemical performance with only the appropriate proportion of melamine. Electrochemical impedance spectroscopy (EIS) revealed enhanced charge transfer kinetics for CFeN‐2 (Figure S, Supporting Information 9) with a lower charge transfer resistance (*R*_ct_), attributed to its reasonable doping atom ratio and micro‐mesoporous structure. Furthermore, the contact angles of CFeN‐1, CFeN‐2, and CFeN‐3 with the electrolyte were also investigated. It is evident that CFeN‐2 showed a smaller angle of 23.5° at the interface compared to NFeC‐1 (32.0°) and CFeN‐3 (36.0°) (Figure S10, Supporting Information). The decrease in contact angle further illustrates the excellent kinetics of CFeN‐2 as a cathode material.

The GCD profiles of CFeN‐2 cathode at various current densities revealed notable reversible capacities of 158, 151, 130, and 120 mAh g^−1^ at 0.5, 1, 2, and 5 A g^−1^, respectively, indicating excellent rate capability (Figure 2c). Additionally, the CFeN‐2 cathode demonstrated stable electrochemical features over 700 cycles, with an initial capacity retention of 95.17% at a current density of 1 A g^−1^ (Figure 2d; Figure S11, Supporting Information). This provides strong evidence of capacity retention and durable cycling stability. Notably, even at a high current density of 10 A g^−1^, the CFeN‐2 cathode exhibited remarkable durability (100 mAh g^−1^ after 10 000 cycles), along with superior structural stability and fast kinetic response. (Figure 2e).

**Figure 3:**
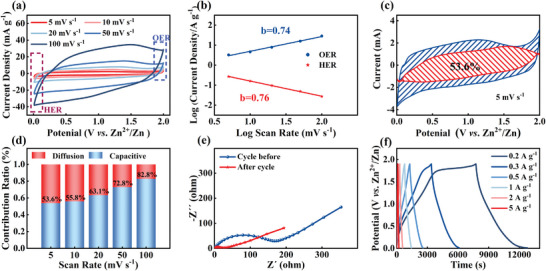
Kinetics analysis of CFeN‐2 cathode. a) CV curves at different scan rates from 1 to 100 mV s^−1^. b) The fitting plots between log(*i*) and log(*v*). c) The capacitive contribution at 5 mV s^−1^ and d) normalized contribution ratios of capacitive capacities at various scan rates. e) Nyquist plots during cycling. f) GCD curves at the current densities of 0.2, 0.3, 0.5, 1, 2, and 5 A g^−1^.

The kinetics parameters of electrochemical reactions and energy storage mechanisms of the constructed Zn//CFeN‐2 system were investigated by CV curves using Dunn's method. **Figure** **3**a shows the cycling voltammetry (CV) curves of CFeN‐2‐based ZICs at 5 mV s^−1^. All the curves exhibit near‐rectangular shapes in the voltage window of 0.1–1.9 V with negligible evidence of oxygen or hydrogen evolution side reactions. Some sub‐structures in the CV curve, for instance, the small cathodic humps at 1.10 V, are reflective of certain redox processes involved.^[^ ^34^, ^35^ ^]^ Moreover, unlike the typical rectangular CV profile observed in electrochemical double‐layer capacitors, the formation of this profile for Zn//CFeN‐2 ZIHCs can be surmised as a combination of diffusion and capacitive processes. The storage mechanism can be revealed by the dependence of the current (i) on the scan rate (v), which obey the following power‐law relationship.^[^ ^36^, ^37^ ^]^

(5) $$i\;=\;a\times v^{b}$$

or convert it to the following formula.^[^ ^38^ ^]^

(6) $$log\;\;\;\left(i\right)=\;b\times \log\left(v\right)+\log\left(a\right)$$

The *b* value is calculated from the slope of the log(*v*)–log(*i*) graph, which can help determine whether the electrochemical reaction is diffusion‐controlled (b = 0.5) or surface reaction‐controlled (b = 1). The b values for CFeN‐2 from charge and discharge curves are 0.74 and 0.76, respectively, indicating that the kinetics of CFeN‐2 are mainly controlled by surface capacitance (Figure 3b). To further verify the capacitive contribution to the current response in the CFeN‐2 cathode, a detailed contribution equation can be quantified using the provided equation.^[^ ^39^ ^]^

(7) $$i\;=k_{1}\;v+k_{2}\;v$$

or convert it to the following formula.

(8) $$i/\;\;\;v^{0.5}=k_{1}\;v^{0.5}+k_{2}$$

where *k*_1_ and *k*_2_ are constants, and *k*_1_ *v* and *k*_2_ *v* ^1/2^ represent the capacitive and diffusion‐controlled contributions of the electrode reactions, respectively. The calculated capacitance contribution was found to be almost 53.6% of the total charge stored at 5 mV s^−1^ for Zn//CFeN‐2 ZIHCs. As the scan rate increased from 5 to 100 mV s^−1^, the capacitive contribution gradually increased from 53.6% to 82.8% (Figure 3c,d). This suggests a dominance of capacitance in charge storage and relatively fast electrochemical kinetics at high scan rates (>10 mV s^−1^), contributing to the excellent rate of electrochemical performance. Furthermore, a diffusion‐dominated mechanism with gradual activation appears at low scan rates due to the unique porous and loosely packed structure of the CFeN‐2 electrode.

Furthermore, the EIS of the samples was investigated before and after cycling, where the semicircle represents the charge transfer resistance (*R*_ct_) and the slope represents the Zn^2+^ ions diffusion resistance (Figure 3e). After cycling, there was a significant decrease in the *R*_ct_ of CFeN‐2 cathode, suggesting enhanced kinetics. Additionally, contact angle measurements were performed on CFeN‐2 both before and after cycling. The contact angle decreased to 10° post‐cycling, indicating enhanced permeability and kinetics subsequent to cycling (Figure S12, Supporting Information). The electrochemical performance of the assembled Zn//CFeN‐2 ZIHC was evaluated within the voltage range of 0.01–1.9 V (Figure 3f). The GCD curves of the ZIHC devices deviated slightly from the linear slope of the “ideal supercapacitor” shape, attributed to the interplay of different energy storage mechanisms in the anode.

**Figure 4:**
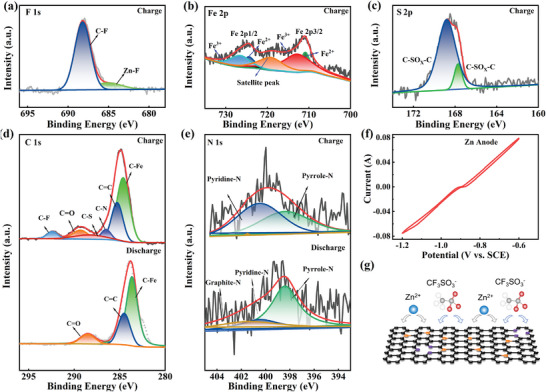
High‐resolution XPS spectrum of a) F 1s, b) Fe 2p, c) S 2p, d) C 1s, and e) N 1s of CFeN‐2 after 100 cycles. f) CV curves of the Zn anode. g) Energy storage mechanism of CFeN‐2 cathode.

X‐ray photoelectron spectroscopy (XPS) analysis was utilized to investigate the energy storage mechanism of CFeN‐2 by analyzing its chemical composition and surface electronic state during full charge and discharge cycles. The comparative survey spectra intuitively demonstrated the successful N doping in CFeN‐2 (Figure S13, Supporting Information). Upon full charge, the two F 1s characteristic peaks at 688.2 and 685.1 eV corresponded to the C─F and Zn─F bonds, respectively (**Figure** **4**a). The Fe 2p spectra exhibited five major peaks, with two peaks centered at 710.9 and 724.4 eV representing the Fe 2p 3/2 and Fe 2p 1/2 signals for Fe^2+^ and two other peaks situated at 712.9 and 726.5 eV associated with the Fe 2p 3/2 and Fe 2p 1/2 signals for Fe^3+^, respectively. Additionally, a satellite peak at 719.2 eV was also observed (Figure 4b).^[^ ^39^ ^]^ Regarding the S species, the S2p peak was designated as two distinct peaks positioned at 167.7 and 168.8 eV, respectively. These peaks represent the C─SO_X_─C (X = 2–4) bond (sulfone bond) in response to the oxidation of the sulfur species,^[^ ^40^ ^]^ which can contribute to pseudo‐capacitance (Figure 4c).

The C1s spectra of CFeN‐2 electrode sheets in the fully charged state (Figure 4d) revealed peaks corresponding to different chemical bonds: C─Fe (283.9 eV), C═C (284.6 eV), C─N (286.1 eV), C─S (287.8 eV), ─COO─Zn (289.0 eV) and C─F (292.1 eV).^[^ ^41^ ^]^ The presence of C─N indicated that nitrogen doping contributes to pseudo‐capacitance. In the N1s spectrum of CFeN‐2 in the fully charged state, two major peaks were observed: pyridine‐N (398.4 eV) and pyrrole‐N (399.5 eV) (Figure 4e). Upon full discharge, the N1s spectra of CFeN‐2 exhibited three peaks: pyridine‐N (398.4 eV), pyrrole‐N (399.5 eV), and graphite‐N (400.5 eV).^[^ ^41^ ^]^ The decrease in pyridine‐N content in the discharged state suggests its role in enhancing capacitance through Faraday redox reaction. Furthermore, graphitic N likely influences the electrical conductivity of the carbon framework by altering its electron‐donor characteristics. Additionally, the existence of N‐containing functional groups in the carbon samples may lead to increased surface defects, providing more electrochemical active sites for Zn^2+^ storage. More importantly, a peak area comparison of different elements in the fully charged and discharged states of CFeN‐2, indicates that the S, F, Zn, and O elements are more in the fully charged state than while in the discharged state (Table S3, Supporting Information). This indicates adsorption and desorption reactions of the electrolyte ion CF_3_SO_3_^−^ on the CFeN‐2 surface during charging and discharging. Figure 4f and Figure S14 (Supporting Information) show the CV curves of the CFeN‐2 cathode and Zn anode in a 3 m Zn(CF_3_SO_3_)_2_ electrolyte. Noteworthy, redox peaks are observed in the range of −0.6–−1.2 V (vs SCE) on the Zn anode, corresponding to the stripping/plating process of Zn^2+^/Zn, whereas CFeN‐2 exhibits pseudocapacitance at higher voltage ranges (−0.2–0.8 V vs SCE). These findings strongly support the anion and cation co‐storage mechanism of the ZlHC system in this study (Figure 4g).

**Figure 5:**
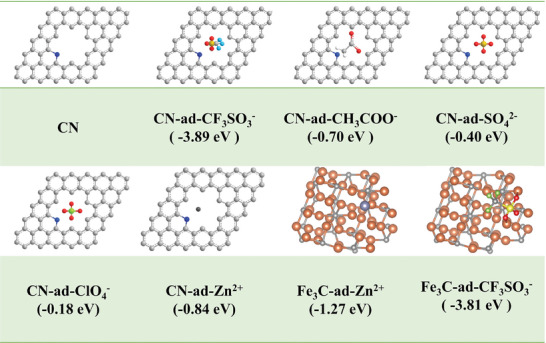
Adsorption of CF_3_SO_3_^−^, CH_3_COO^−^, SO_4_^2−^, ClO_4_^−^, Zn^2+^ ions on pyridine N‐doped porous carbon and Fe_3_C, respectively.

To further elucidate the specific role of N atoms in the adsorption/desorption process of anions and cations at the CFeN‐2 interface, the relative adsorption energy (*ΔEa*) values of CF_3_SO_3_^−^, CH_3_COO^−^, SO_4_^2−^, ClO_4_^−^ anions and Zn^2+^ cation at the N atom heterogeneous site and the defect site were calculated, respectively. Specifically, using pyridine‐N as the structural model, each CF_3_SO_3_^−^, CH_3_COO^−^, SO_4_^2−^, and ClO_4_^−^ anion was positioned near each the respective site, and the optimized geometry was determined based on the calculated *ΔE_a_*. The results, shown in **Figures** **5** and S15 (Supporting Information), reveal that the *ΔE_a_* values for CF_3_SO_3_^−^, CH_3_COO^−^, SO_4_^2−^, and ClO_4_^−^ anions adsorption at pyridine‐N are −3.89, −0.70, −0.40, and −0.18 eV, respectively, indicating the active role of pyridine‐N in anion adsorption, with CF_3_SO_3_^−^ anion exhibiting the most negative adsorption energy. Additionally, the *ΔE_a_* values between CF_3_SO_3_^−^, Zn^2+^ ions and Fe_3_C were also calculated. The results show that Fe_3_C exhibits a lower adsorption energy for Zn^2+^ ions (−1.27 eV vs −0.84 eV), demonstrating the zincophilic nature of Fe_3_C. Furthermore, the *ΔE_a_* values of CF_3_SO_3_^−^ anion on Fe_3_C (−3.81 eV) closely resemble those on CN (−3.89 eV), underscoring the strong adsorption capability of CFeN‐2 material toward CF_3_SO_3_^−^ and Zn^2+^ ions.

Moreover, the effects of four typical zinc salts, namely Zn(CH_3_COO)_2_, ZnSO_4_, Zn(ClO_4_)_2_, and Zn(CF_3_SO_3_)_2_ on the electrochemical performance were also comparatively analyzed. The results indicated that the Zn//CFeN‐2 ZIHC using Zn(CF_3_SO_3_)_2_ electrolyte exhibited the highest and most stable discharge‐specific capacity. This suggests the unique and stable ability of CFeN‐2 cathode to absorb and desorb Zn^2+^ and CF_3_SO_3_^−^ ions, thereby achieving a high‐capacity and long‐cycle ZIHC (Figure S16a, Supporting Information). The storage mechanism of CFeN‐2 cathode may be illustrated by Equations (9) and (10).^[^ ^41^ ^]^ Besides, the impact of electrolyte concentration on the electrochemical performance of CFeN‐2 was investigated. The capacity retention rates were 85.73%, 86.68%, and 99.59% after 640 cycles with 1–3 m Zn(CF_3_SO_3_)_2_ electrolytes at a current density of 1 A g^−1^, with the CE enhancing with the increase of electrolyte concentration (Figure S16b, Supporting Information). It is important to note that concentrations of 4 M or higher were not tested due to solubility limitations.

(9) $$\mathrm{Anode}:\;Zn^{2+}+2e^{-}↔\mathrm{Zn}$$

(10) $$\begin{array}{ccc} & & \mathrm{Cathode}:\;C+CF_{3}{\mathrm{SO}}_{3}^{-}↔C\left|\right|CF_{3}{\mathrm{SO}}_{3}^{-}\\ & & -C=O-\mathrm{OH}+Zn^{2+}+e^{-}↔-C=O-O-\mathrm{Zn}+H^{+} \end{array}$$

**Figure 6:**
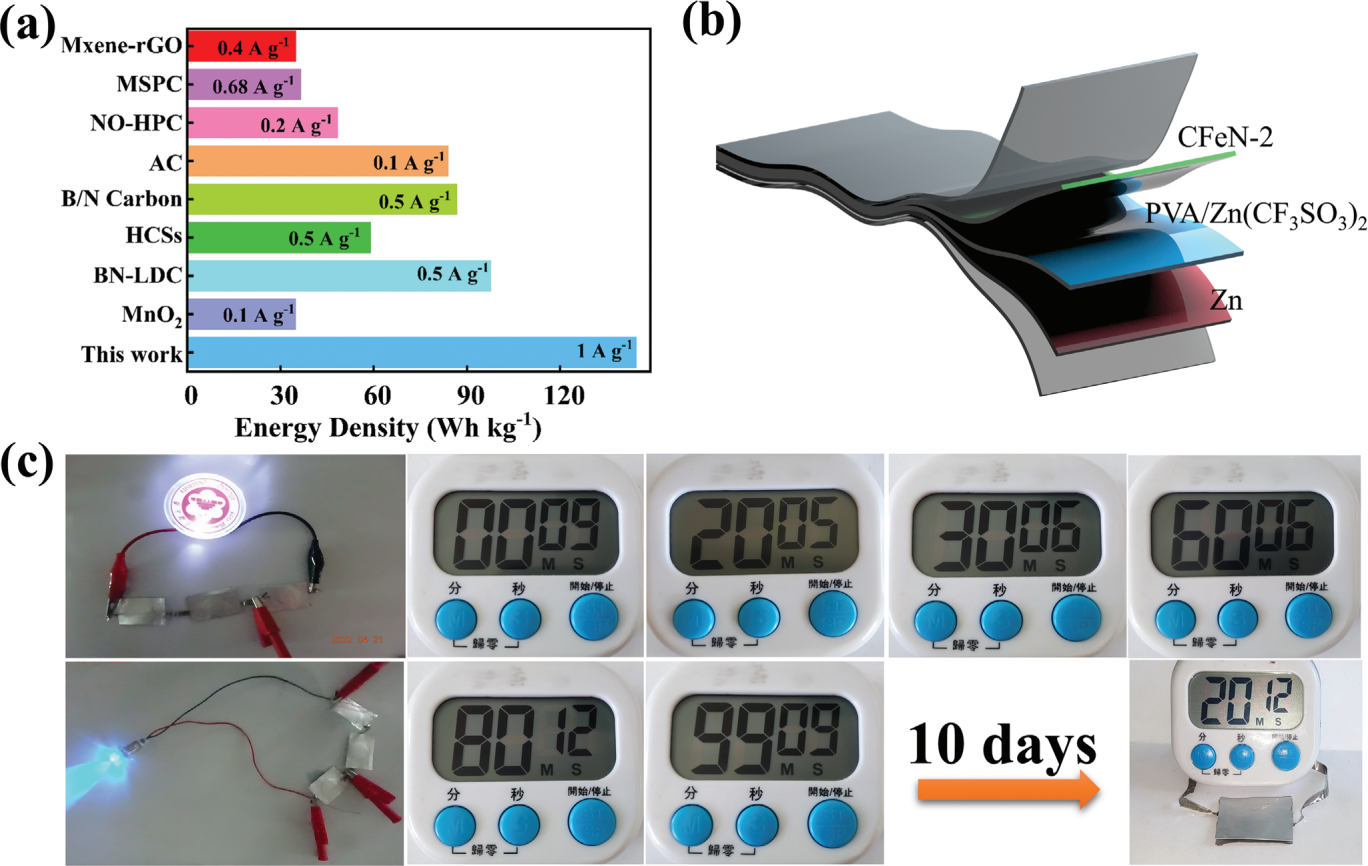
a) Comparison of the energy density of CFeN‐2 cathode with other cathodes in ZIHCs. b) CFeN‐2‐based quasi‐solid‐state ZIHCs. c) Photograph of CFeN‐2 cathode in quasi‐solid‐state ZIHCs lighting up a small bulb and powering a timer at different discharge times.

Comparative analysis of cycling performance, energy density, and power density against other capacitor cathode materials indicates that CFeN‐2 exhibits superior electrochemical performance (Tables S4 and S5, Supporting Information). The Zn//CFeN‐2 ZIHCs delivered high energy density and high power output, achieving a maximum energy density of 142.5 W h k g^−1^ at a power density of 951.8 W kg^−1^. Even at a substantial power density of 9500.1 W kg^−1^, the energy density remains at 95.1 W h kg^−1^, which is considerably better than the recently reported carbon cathode‐based ZIHCs (**Figure** **6**a). In addition, to estimate the potential of CFeN‐2 cathode in flexible energy storage devices, a quasi‐solid‐state ZIHC was assembled using a Zn(CF_3_SO_3_)_2_/PVA gel electrolyte (Figure 6b). The Zn//CFeN‐2 system successfully powered LED lamps. In addition, two ZIHC devices were connected in series to power an electronic timer continuously for 10 days without interruption. These findings demonstrate that CFeN‐2 as a cathode in quasi‐solid‐state ZIHC showcases great potential for applications in flexible electronics (Figure 6c).

## 3 Conclusion

In conclusion, we demonstrated a novel carbon cathode with high performance for ZIHCs using coal as a resource. By leveraging the synergistic effects of FeCl_3_ catalytic pore creation and nitrogen doping of melamine, the advanced CFeN‐2 cathode demonstrated a high specific surface area, graded porous structure, and abundant nitrogen doping. These unique characteristics enhance sufficient ion/charge storage capacity and promote fast ion/electron transfer rates. Theoretical and experimental findings suggest that CFeN‐2 exhibits excellent electrochemical performance in both Zn(CF_3_SO_3_)_2_ aqueous solution and PVA//Zn(CF_3_SO_3_)_2_ gel electrolyte through a dual‐ion energy storage mechanism involving Zn^2+^ ions and CF_3_SO_3_^−^ anions, making it a promising candidate for ZIHCs applications. The Zn//CFeN‐2 ZIHCs device achieved a remarkable energy density of 142.5 Wh kg^−1^ and a high‐power output of 9500.1 W kg^−1^, along with a long cycle life of 10000 cycles. This research presents a novel design approach for constructing high‐performance, safe, and eco‐friendly ZIHCs.

## Conflict of Interest

The authors declare no conflict of interest.

## Supporting information



Supporting Information

## Data Availability

The data that support the findings of this study are available from the corresponding author upon reasonable request.
