# Supplementary material for: N‐Doped Porous Carbon Based on Anion and Cation Storage Chemistry for High‐Energy and Power‐Density Zinc Ion Capacitor
Source: Adv Sci (Weinh). 2024 Oct 7;11(44):2407635. doi: 10.1002/advs.202407635 (PMC11600265; doi:10.1002/advs.202407635)
Supplement: Supplementary file 1 — Supporting Information [file ADVS-11-2407635-s001.docx]

**Supporting Information**

**N-doped Porous Carbon Based on Anion and Cation Storage Chemistry for High-Energy and Power-Density Zinc Ion Capacitor**

Yuanyuan Liang^a#^, Miaomiao Wu^a#^, Anjie Liu^a*^, Qihua Chen^a^, Yan Wu^a^, Qian Xiang^a^, Zhibo Liu^a^, Jixi Guo^a^, Xingchao Wang^ab*^ and Dianzeng Jia^a*^

Y. Liang, M. Wu, A. Liu, Q. Chen, Y. Wu, Q. Xiang, Z. Liu, J. Guo, X. Wang, D. Jia

^a^State Key Laboratory of Chemistry and Utilization of Carbon Based Energy Resources; Key Laboratory of Advanced Functional Materials, Autonomous Region; Institute of Applied Chemistry, College of Chemistry, Xinjiang University, Urumqi, 830046, Xinjiang, P. R. China

X. Wang

^b^Key Laboratory of Advanced Energy Materials Chemistry (Ministry of Education), Nankai University, Tianjin 300071, China

E-mail: [liuanjie@xju.edu.cn](mailto:liuanjie@xju.edu.cn); [ichemabc@126.com](mailto:ichemabc@126.com); jdz@xju.edu.cn

^#^Yuanyuan Liang and Miaomiao Wu contributed equally to this work.

**Experimental section**

1. ***Materials***

FeCl_3_·6H_2_O (98%) and melamine (99%) were ordered from Shanghai Macklin Biochemical Co., Ltd. polyvinylidene fluoride (PVDF), 1-Methyl-2-pyrrolidinone (NMP), and Super P were purchased from Sinopharm Chemical Reagent Co., Ltd. All chemicals were used directly without any further treatment.

1. ***Synthesis of Materials***

***2.1 Synthesis of CN samples***

Coal pitch and melamine were mixed evenly in a mortar with mass ratio of 1:1, 1:2, 1:3 and 1:4. The same preparation method as for the synthesis of CN-x was used. The prepared carbon materials were denoted as CN-x (X=1, 2, 3, 4).

***2.2 Synthesis of CFe samples***

Coal pitch and FeCl_3_·6H_2_O were mixed in a mortar at the weight ratio of 1:9, 1: 11, 1:13 and 1:15. The mixed powders were wrapped with carbon paper, heated in an N_2_ filled tube furnace at 800°C for 3 h with a ramp rate of 5℃ min^-1^, then cooled down to room temperature. Next, the calcined samples were dispersed in 50 mL of 1 M HCl aqueous solution to remove the residual Fe, and then washed with deionized water to obtain samples. The prepared materials were denoted as CFe-x (X=1, 2, 3, 4).

***2.3 Synthesis of CFeN-x samples***

Coal pitch, melamine and FeCl_3_·6H_2_O were mixed evenly in a mortar at the weight ratio of 1: 11: 1, 1: 11: 2, 1: 11: 3 and 1: 11: 4. The mixtures were firstly heated to 550°C (decomposition temperature of melamine) with a heating rate of 5°C min^−1^ for 2 hours under nitrogen atmosphere, then heated continuously to 800℃ with 5℃ min^-1^ for 2 hours. After natural cooling, the calcined samples were dispersed in 50 mL of 1 M aqueous hydrochloric acid solution and stirred for 6 h. Finally, the mixtures were purified by distilled water and dried under vacuum at 80 ℃ for 24 h. The as-obtained sample were named as CFeN-X (the X represents the number of 1, 2, 3, and 4).

***3. Material characterizations***

The microscopic morphology of the materials was observed with a scanning electron microscope (SEM, Hitachi SU-4800, Japan) and transmission electron microscope (TEM, JEOL, JEM-2100F, Japan). The chemical properties of the material surface were performed by Energy dispersive spectroscopy (EDS). The molecular structure of the materials was characterized by. Fourier transform infrared (FTIR, Bruker VERTEX 70, Germany). The phase structures were measured by X-ray diffractometer (XRD, BRUKER D8 Advanced, Germany). The atomic composition of the sample surface was determined by X-ray photoelectron spectroscopy (XPS, Thermo Fisher, Escalab 250, USA). Raman spectra were recorded by using a Raman spectrometer (LabRAM HR Raman Instrument). The specific surface area and pore characteristic were collected by the N_2_ adsorption analyzer (Micromeritics ASAP 2020).

***4. DFT calculations***

All calculations on density generalized function theory (DFT) were performed using the VASP (Vienna ab initio simulation package) software. In which the Perdew-Burke-Ernzerhof (PBE) generalized gradient approximation (GGA) and the projection augmented wave (PAW) method were used. The valence/outer-core electrons that are included in the self-consistent-field calculations are listed inter parentheses for each atom: C(2s^2^ 2p^2^), N(2s^2^ 2p^3^), O(2s^2^ 2p^4^), F(2s^2^ 2p^5^), S(3s^2^ 3p^4^), Cl(3s^2^ 3p^5^) and H(1s^1^). For all optimization calculations, the cutoff energy was set to 420 eV, K-point was set to 3*3*1. The convergence criteria for energy and force were set to 10^-5^ eV and 0.01 eV Å^-1^. A 5 × 5 monolayer graphene supercell was constructed to simulate graphene, and a 12 Å vacuum layer was constructed to prevent interactions between periodic layers at 18 Å. The adsorption energies (E_ads_) of ions on graphene were calculated as follows.

*E*_ads_ = *E*_total_ − *E*_surface_ – *E*_species_

*E*_total_, *E*_surface_ and *E*_species_ represent the total energy of the adsorbed species on the graphene surface, the energy of the empty graphene surface and the gas phase energy of the specific adsorbed species.

***5. Electrochemical characterizations***

For fabrication of the working electrodes, 80 wt% of active material, 10 wt% of acetylene black and 10 wt% of polyvinylidene fluoride (PVDF) in methyl-2-pyrrolidone (NMP) were thoroughly mixed, and then coated on the current collectors (Ti foils), and were finally dried in a vacuum oven at 110 ℃ for 12 h before use. After drying, and punched into disks with a diameter of 12 mm as the test electrode. Using zinc flakes as the anode (to make sure that the surface is free of zinc oxide, zinc flakes was soaked in anhydrous ethanol: acetone = 1:1 for ultrasonic half an hour, then soaked in anhydrous ethanol for ultrasonic half an hour), glass microfiber membrane (GF/D, Whatman) as the separator, 3 mol L^-1^ Zn(CF_3_SO_3_)_2_ as the electrolyte and CFeN-2 as the cathode to assemble button hybrid capacitor device (CR2016).

The cyclic voltammograms (CV) of hybrid zinc ion capacitor was measured by electrochemical workstation (CHI760E). The cycle performance of the electrode was tested with the Land electrical test software. The test process was carried out in the voltage range of 0.01−1.9 V. The specific capacitance of the battery is calculated by equation (1) below:

C = 2Iꭍ V dt / 3.6Vm (mAh g^-1^) (1)

Where I (A) represents the discharge current (A), ꭍ V dt (V) represents the integrated area under the discharge curve, V (V) represents the voltage after the ohmic drop, m (g) is the mass of the cathode active material.

The energy density of the battery can be calculated from the specific capacitance (C, mAh g^-1^) and the battery discharge voltage (V), the formula is as follows:

E = CV/2 (Wh·kg^-1^) (2)

Power density (P) can be calculated by energy density (E) and discharge time (t), the formula is as follows:

P = E × 3600/t (W·kg^-1^) (3)


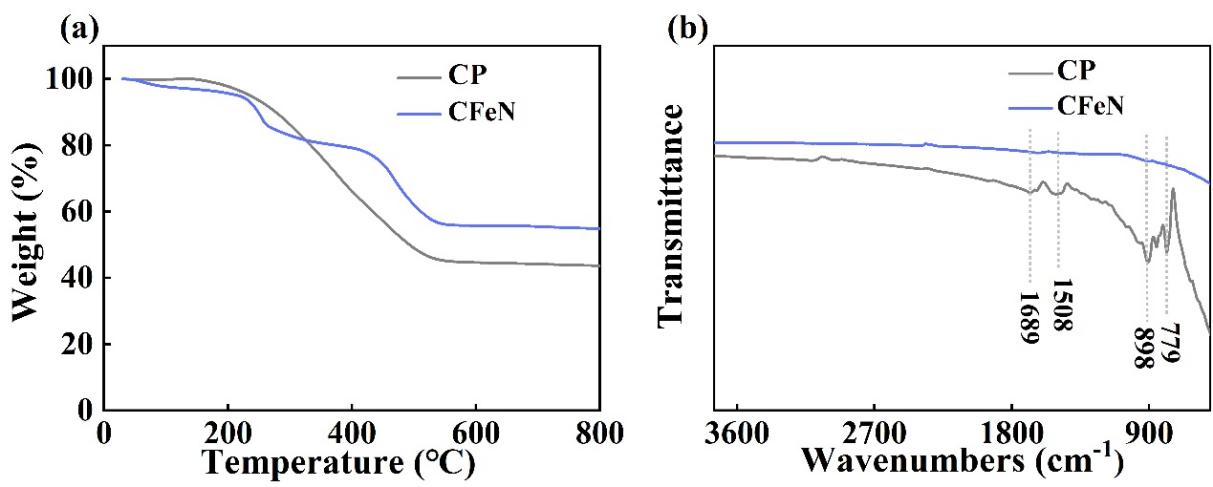


**Figure S1.** (a) TG curves and (b) FTIR spectra of CP and CFeN.


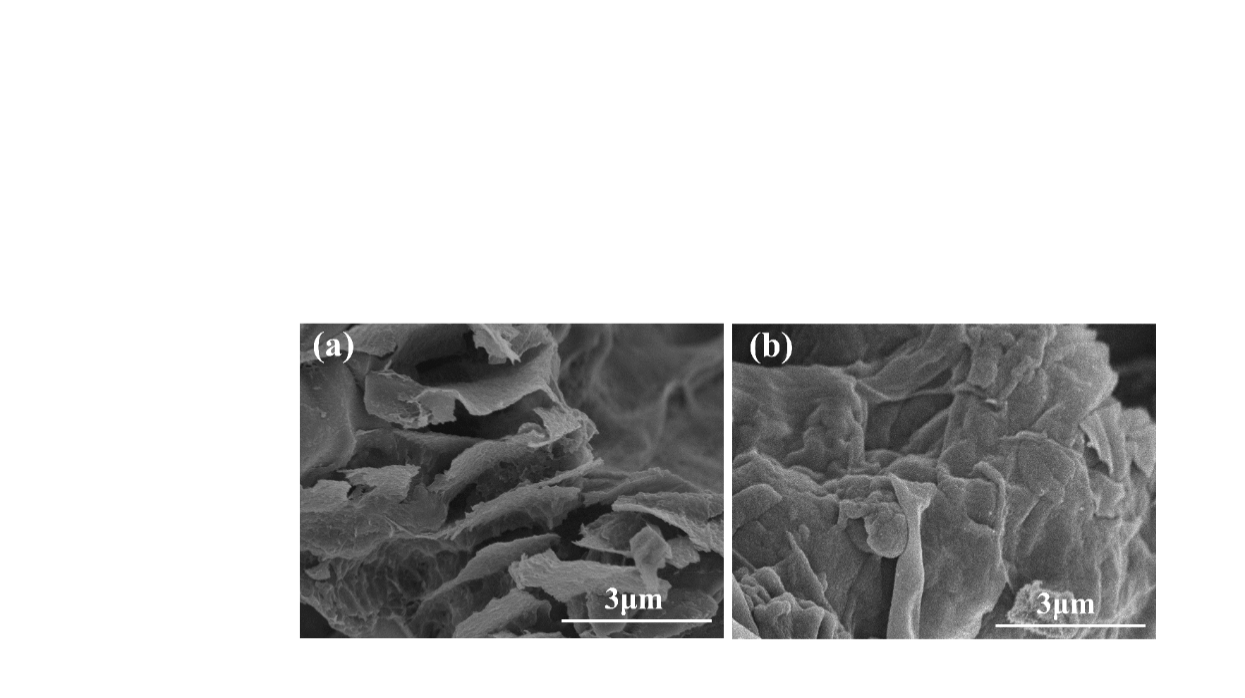


**Figure S2.** SEM images of (a) CFeN-2 and (b) CFeN-3.


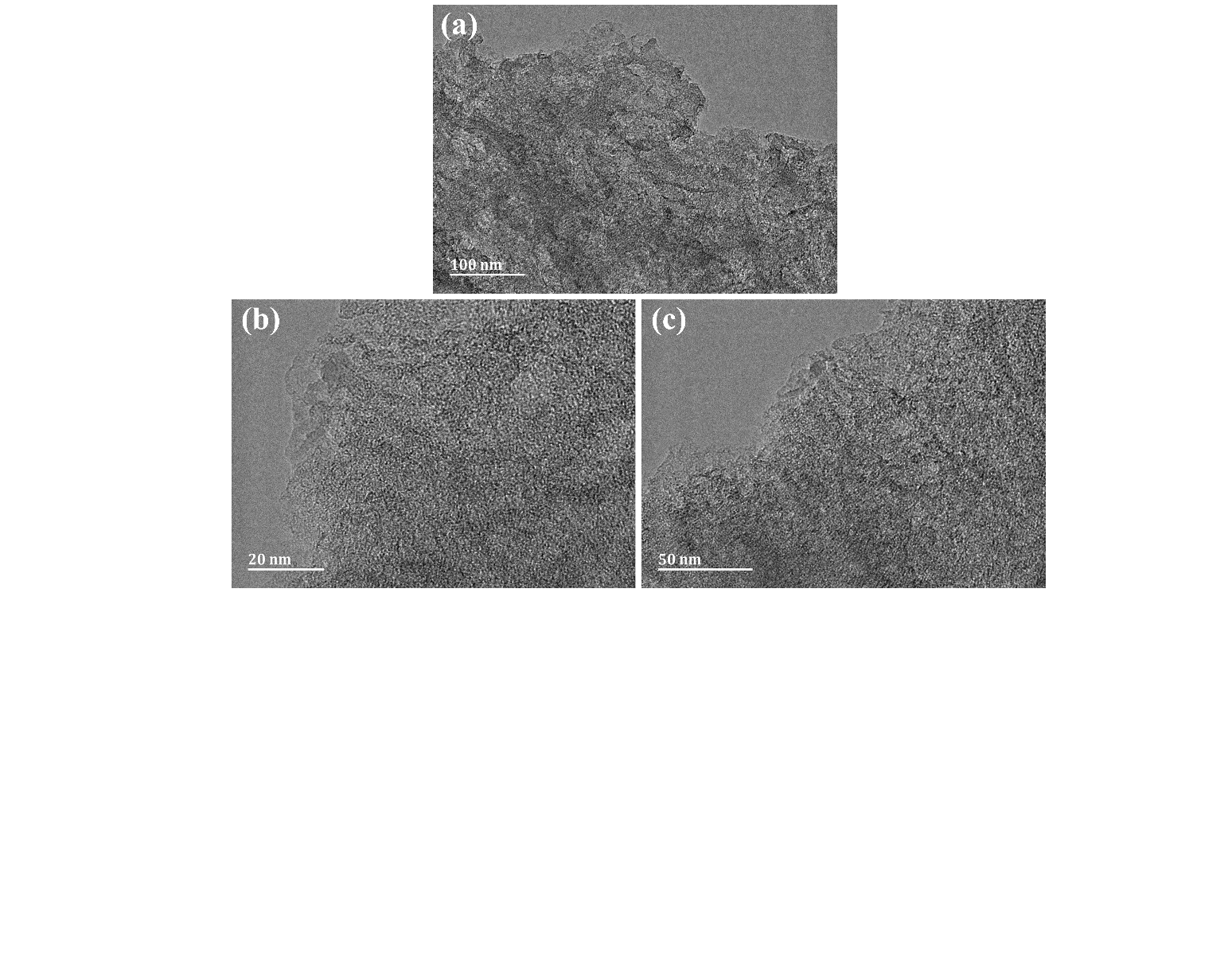


**Figure S3.** HRTEM images of CFeN-2.


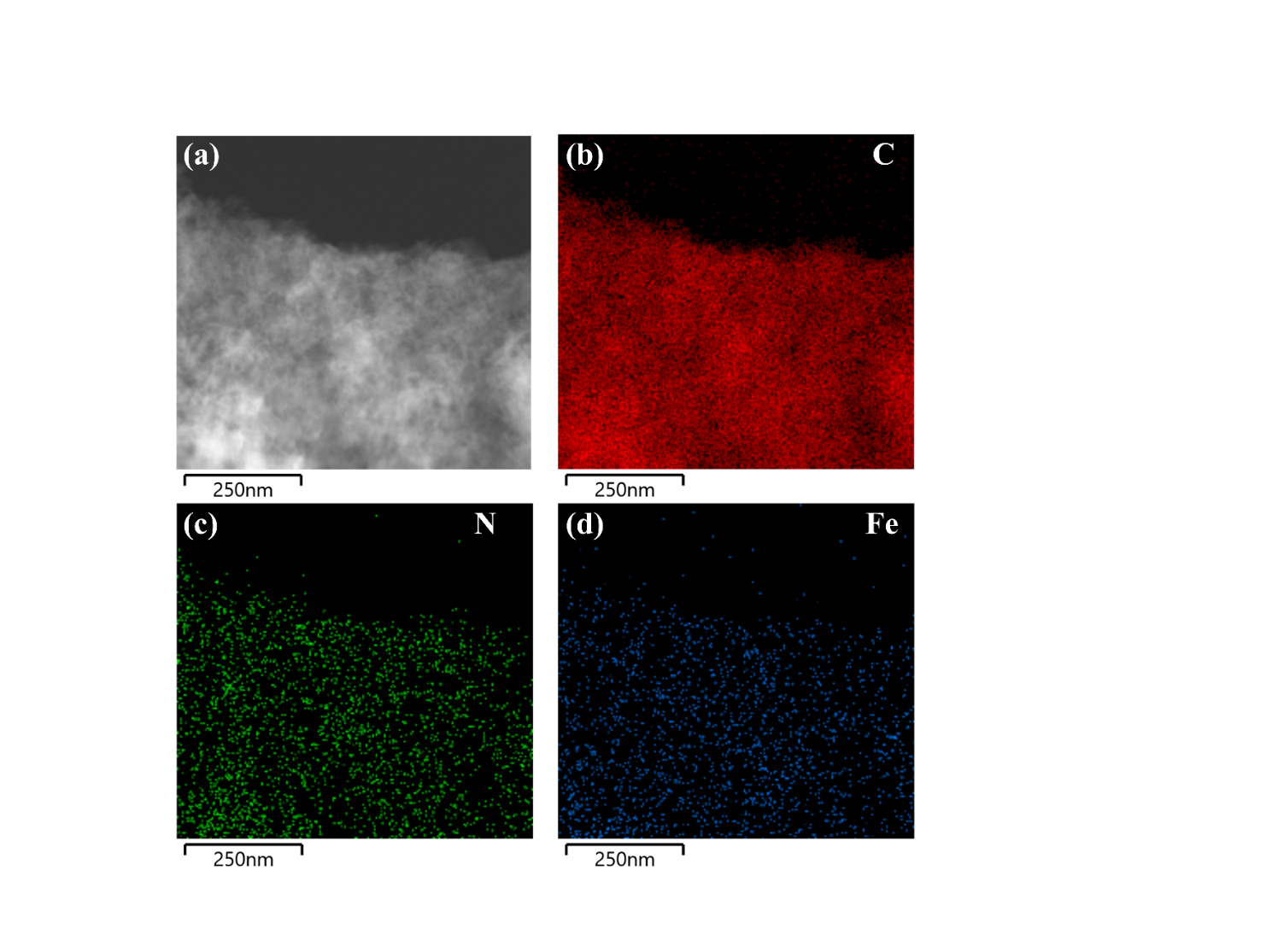


**Figure S4.** (a) HRTEM images and (b-d) mapping images of CFeN-2.


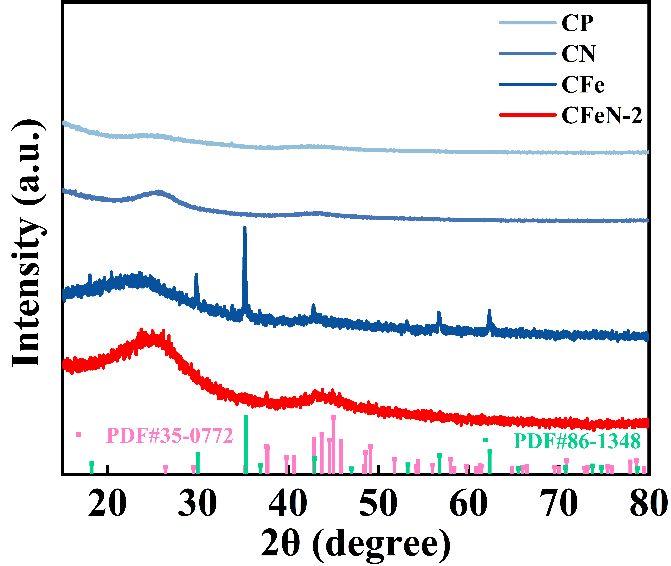


**Figure S5.** XRD patterns of CP, CN, CFe and CFeN-2.


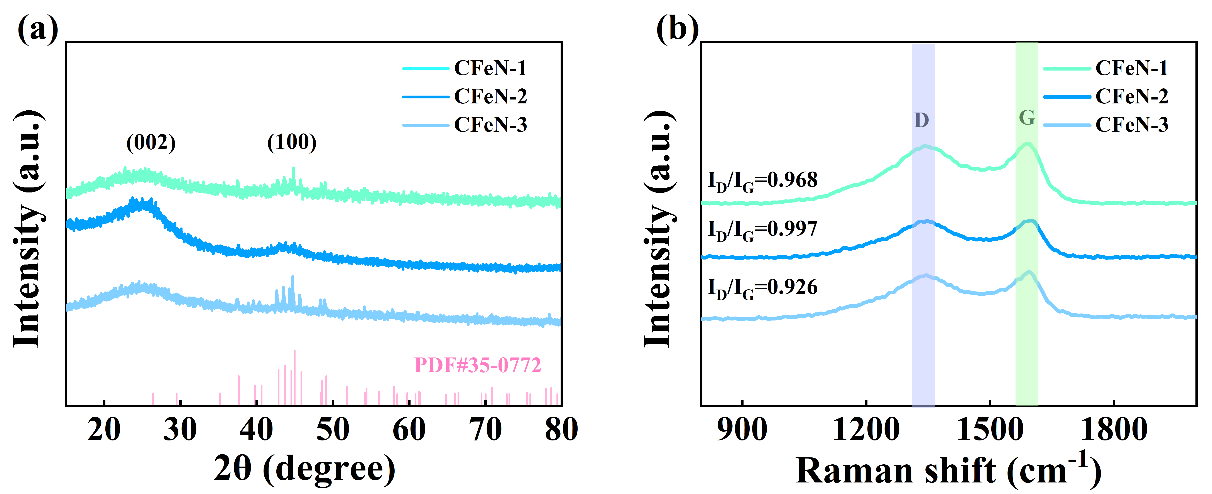


**Figure S6.** (a) XRD patterns, and (b) Raman spectra of CFeN-1, CFeN-2, CFeN-3.


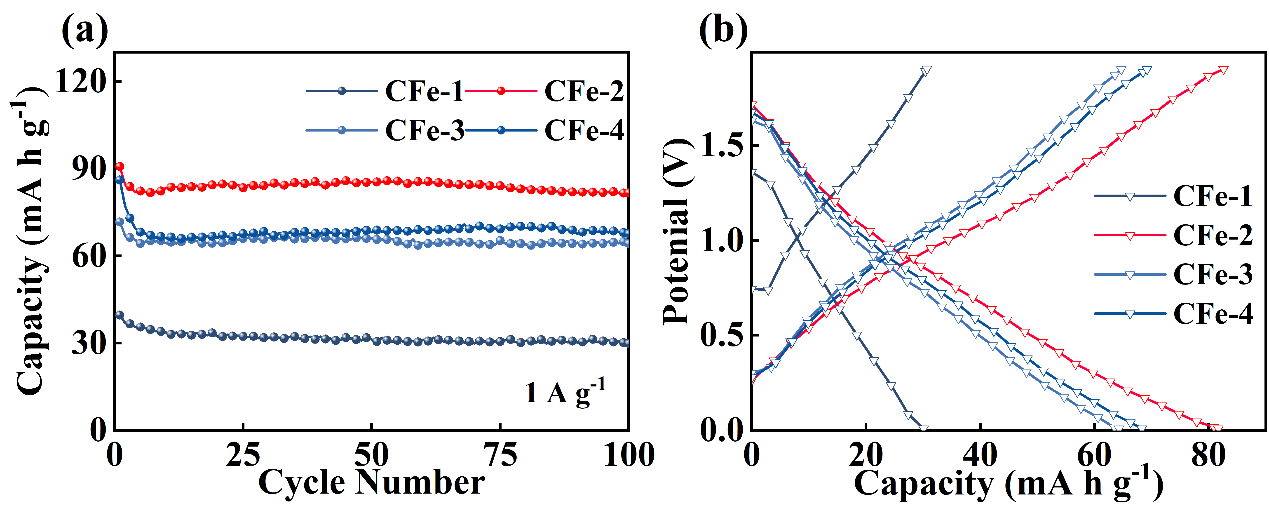


**Figure S7.** (a) Discharge specific capacity and (b) Charge-discharge curves of CFe-1, CFe-2, CFe-3 and CFe-4 cathodes at the current density of 1 A g^−1^, respectively.


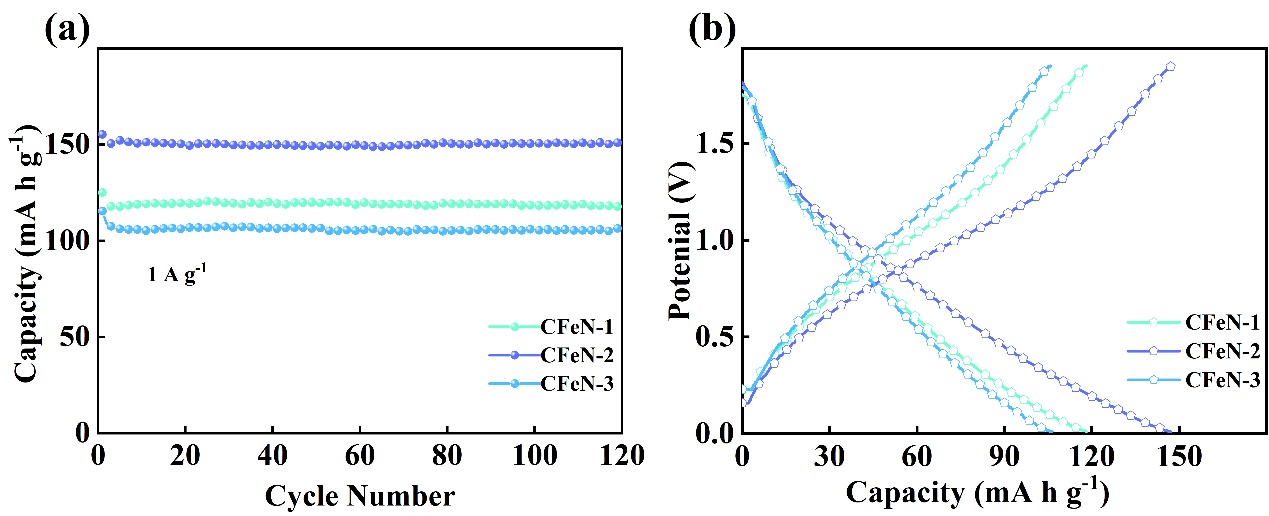


**Figure S8.** (a) Discharge specific capacity and (b) Charge-discharge curves of CFeN -1, CFeN -2 and CFeN -3 cathodes at the current density of 1 A g^−1^.


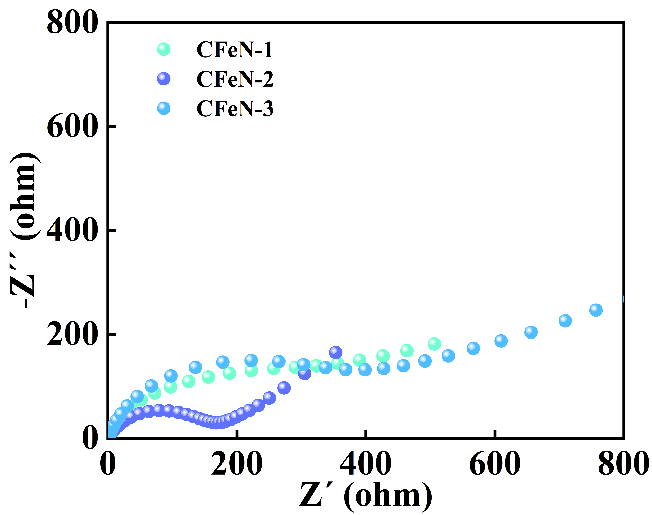


**Figure S9.** EIS of (a) CFeN-1, (b) CFeN-2 and (c) CFeN-3.


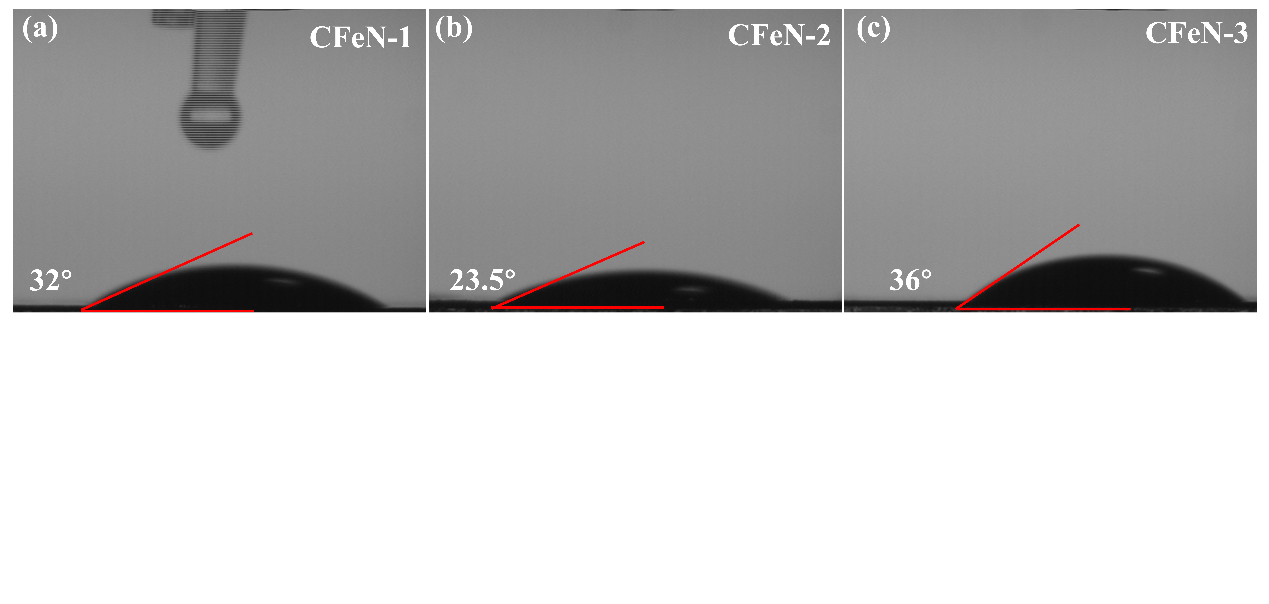


**Figure S10.** Contact angle of electrolyte on (a) CFeN-1, (b) CFeN-2, (c) CFeN-3 cathodes, respectively.


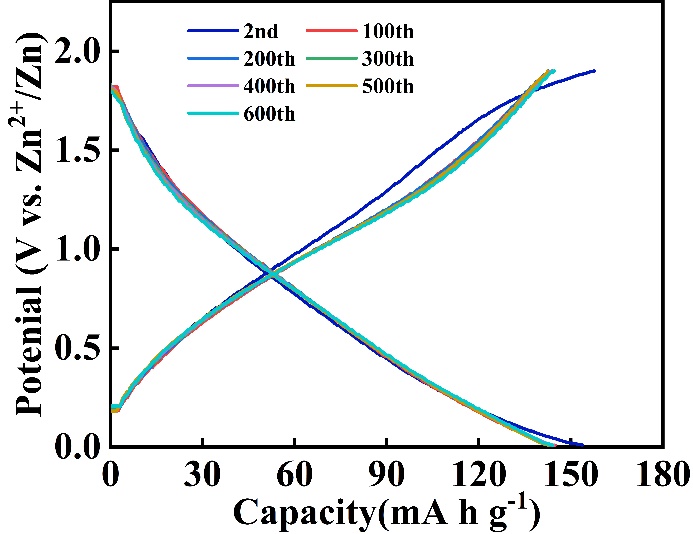


**Figure S11.** GCD curves of the Zn//CFeN-2 ZIHC at the 2nd, 100th, 200th, 300th, and 400th cycles at 1 A g^-1^, respectively.


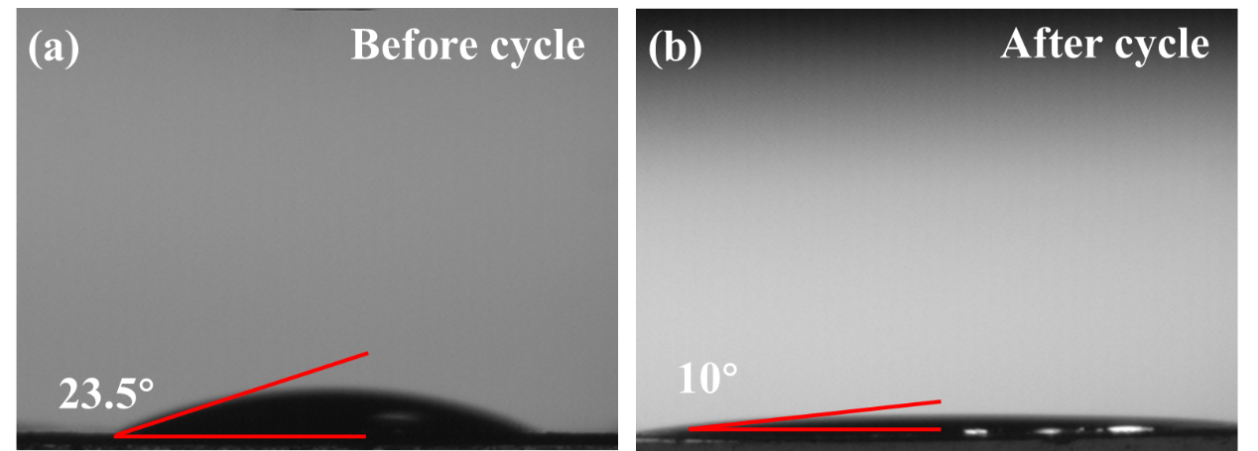


**Figure S12**. Contact angle of CFeN-2 at (a) before cycle and (b) after cycle, respectively.


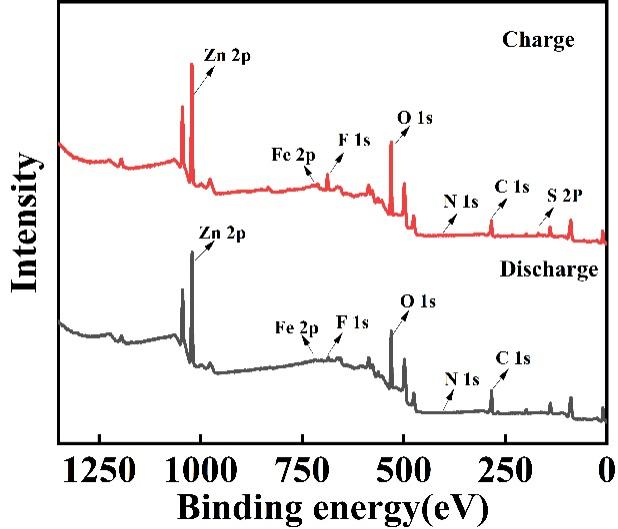


**Figure S13.** The XPS survey spectrum of charge and discharge XPS of CFeN-2 after 100 cycles.


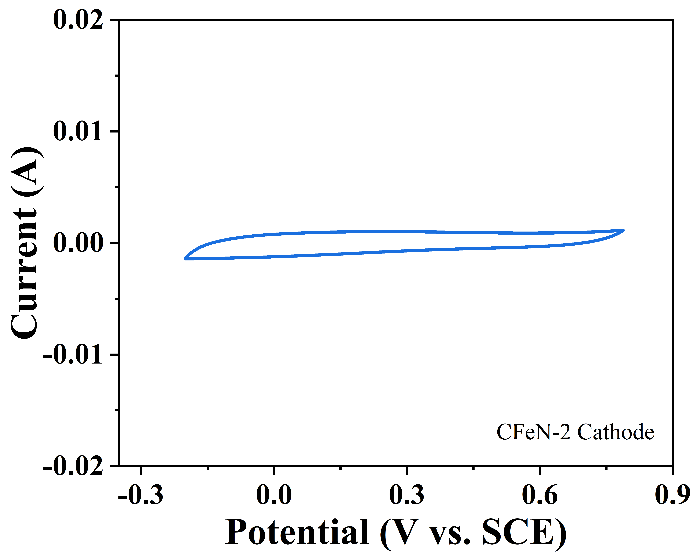


**Figure S14.** CV curves of the CFeN-2 cathode.


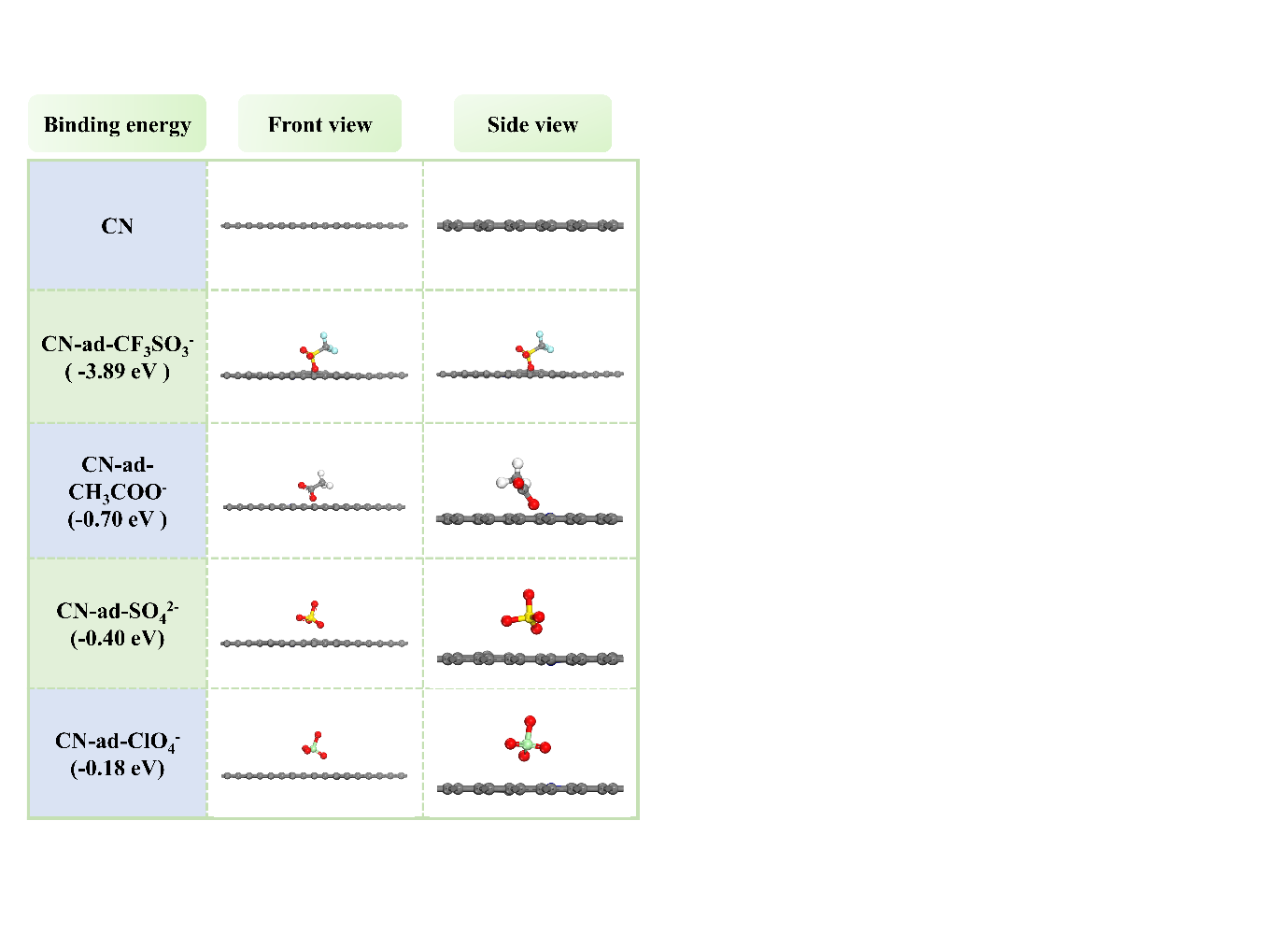


**Figure S15.** Front and side views of adsorption of CF_3_SO_3_^-^, CH_3_COO^-^, SO_4_^2-^, ClO_4_^-^ anions on pyridine N-doped porous carbon, respectively.


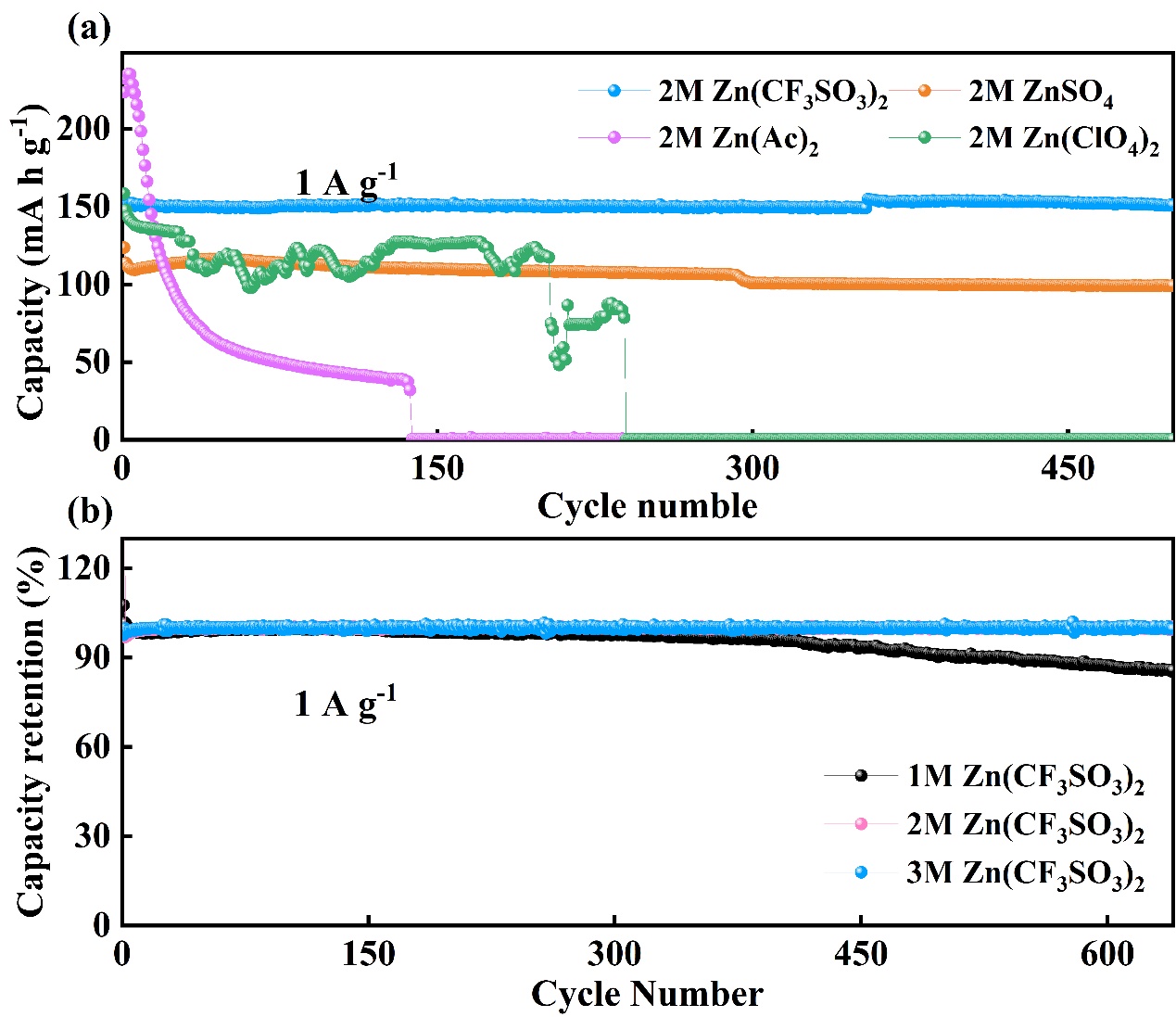


**Figure S16.** Cycling performance of CFeN-2 cathode in (a) different electrolytes and (b) different electrolyte concentrations.

**Table S1.** Elemental contents of two Samples.

| Samples | Pitch | | CFeN-2 | |
| --- | --- | --- | --- | --- |
| Element | *W* t % | *A* t % | *W* t % | *A* t % |
| *C*K | 97.51 | 97.94 | 85.63 | 89.42 |
| *N*K | 02.36 | 02.04 | 10.95 | 09.81 |
| *Fe*K | 00.13 | 00.03 | 03.42 | 00.77 |

**Table S2.** Pore structure parameters of CP, CN, CFe and CFeN-2 samples.

| Samples | Samples D_ap_ (nm) | S_BET_  (m^2^ g^-1^) | S_mic_  (m^2^ g^-1^) | V_t_  (cm^3^ g^-1^) | V_mic_  (cm^3^ g^-1^) |
| --- | --- | --- | --- | --- | --- |
| CP | 7.231 | 2.028 | 4.513 | 0.003 | 0.0002 |
| CN | 17.116 | 32.915 | 4.513 | 0.140 | 0.0010 |
| CFe | - | 1182.45 | - | - | - |
| CFeN-2 | - | 1037.66 | - | - | - |

*D*_ap_: average pore size;

*S*_BET_: specific Brunauer-Emmett-Teller surface area;

*S*_mic_: micropore surface area;

*V*_t_: total pore volume;

*V*_mic_: micropore volume.

**T****able S3.** XPS peak areas of CFeN-2 after discharge and charge cycles.

| Element | Zn | O | F | S |
| --- | --- | --- | --- | --- |
| Peak area of charge | 660747 | 202430 | 43713 | 6761 |
| Peak area of discharge | 605978 | 159185 | 13212 | 683 |

**Table S4.** Comparison of cycle performance of **CFeN-2 with other carbon-based cathodes**.

| **Cathode** | **Anode** | **Capacity (mAh g^-1^)** | **Current (A g^-1^)** | **Ref** |
| --- | --- | --- | --- | --- |
| PGCNs | Zn foil | 133.2 | 1.0 | ^1^ |
| Zn-MET800 | Zn foil | 110.2 | 1.0 | ^2^ |
| N-HPC | Zn foil | 136.8 | 0.1 | ^3^ |
| LDC | Zn foil | 127.7 | 0.5 | ^4^ |
| OPC | Zn foil/Carbon cloth | 132.7 | 1.0 | ^5^ |
| AC | Zn foil | 121.0 | 0.1 | ^6^ |
| MnO_2_ nanorods | AC | 46.4 | 0.1 | ^7^ |
| HCS | Zn/Carbon cloth | 86.8 | 0.5 | ^8^ |
| NO-BPC | Zn/CC | 51.4 | 0.2 | ^9^ |
| Carbon nanosheets | Zn foil | 90.0 | 0.1 | ^10^ |
| S-doped 3D porous carbons | Zn foil | 123.8 | 0.2 | ^11^ |
| **CFeN-2** | **Zn foil** | **150.0** | **1.0** | **This work** |

**Table S5.** Energy density and power density comparisons of **CFeN-2 with other carbon-based cathodes**.

| **Cathode** | **Anode** | **Energy density (Wh kg^-1^)** | **Power density (W kg^-1^)** | | | **Ref** |
| --- | --- | --- | --- | --- | --- | --- |
| PZC-10 | CC@ZIF-8/Zn foil | 110.0 | | 500.0 | ^12^ | |
| GROC-A13 | Zn foil | 116.0 | | 800.0 | ^13^ | |
| RHCs | Zn foil | 58.6 | | 167.8 | ^14^ | |
| CMF-800 | Zn foil | 54.0 | | 14650.0 | ^15^ | |
| PC | Zn foil | 67.4 | | 152.9 | ^16^ | |
| oxygen-rich porous carbon | Zn foil | 104.8 | | 4880.0 | ^17^ | |
| SS-0.7 | Zn foil | 87.6 | | 36.9 | ^18^ | |
| PN-CHoNS | Zn\|In | 116.0 | | 141.0 | ^19^ | |
| MCHSs | Zn foil | 129.3 | | 266.4 | ^20^ | |
| HPCS-900 | Zn foil | 90.2 | | 81.2 | ^21^ | |
| N-OPCNF | Zn foil | 98.3 | | 72.3 | ^22^ | |
| SN-PCNTs | In@Zn | 95.9 | | 125.0 | ^23^ | |
| Ca-900 | Zn foil | 75.2 | | 36.6 | ^10^ | |
| PCNs-2 | Zn foil | 60.0 | | 15.9 | ^24^ | |
| PCNF-4 | Zn foil | 102.1 | | 16.9 | ^25^ | |
| **CFeN-2** | **Zn foil** | **142.5** | | **951.8** | **This work** | |
| **CFeN-2** | **Zn foil** | **95.1** | | **9500.1** | **This work** | |

**References**

1. Yang Y, Ni G, Liu L, Zhao J, Qu S. Designing P-doped graphite-like hierarchical porous carbon nanosheets from coal tar pitch for enhanced Zn-ion hybrid capacitors. *Colloids Surf., A.* 2024; *695*.

2. Jia D, Shen Z, Zhou W, et al. Ultrahigh N-doped carbon with hierarchical porous structure derived from metal-organic framework for high-performance zinc ion hybrid capacitors. *Chem. Eng. J.* 2024; *485:*149820.

3. Liu P, Gao Y, Tan Y, et al. Rational design of nitrogen doped hierarchical porous carbon for optimized zinc-ion hybrid supercapacitors. *Nano Research.* 2019; *12* (11):2835-2841.

4. Lu Y, Li Z, Bai Z, et al. High energy-power Zn-ion hybrid supercapacitors enabled by layered B/N co-doped carbon cathode. *Nano Energy.* 2019; *66:*104132.

5. Zheng Y, Zhao W, Jia D, Liu Y, Liu J. Porous carbon prepared via combustion and acid treatment as flexible zinc-ion capacitor electrode material. *Chem. Eng. J.* 2020; *387*:124161.

6. Dong L, Ma X, Li Y, et al. Extremely safe, high-rate and ultralong-life zinc-ion hybrid supercapacitors. *Energy Storage Mater.* 2018; *13:*96-102.

7. Ma X, Cheng J, Dong L, et al. Multivalent ion storage towards high-performance aqueous zinc-ion hybrid supercapacitors. *Energy Storage Mater.* 2019; *20* (335)-342.

8. Chen S, Ma L, Zhang K, Kamruzzaman M, Zhi C, Zapien J A. A flexible solid-state zinc ion hybrid supercapacitor based on co-polymer derived hollow carbon spheres. *J Mater Chem A.* 2019; *7* (13):7784-7790.

9. Chen H, Zheng Y, Zhu X, et al. Bamboo-derived porous carbons for Zn-ion hybrid supercapacitors. *Materials Research Bulletin.* 2021; *139:*111281.

10. Zhang Y, Wang Z, Li D, et al. Ultrathin carbon nanosheets for highly efficient capacitive K-ion and Zn-ion storage. *J. Mater. Chem. A.* 2020; *8* (43):22874-22885.

11. Wang D, Wang S, Lu Z. S‐doped 3D porous carbons derived from potassium thioacetate activation strategy for zinc‐ion hybrid supercapacitor applications. *International Journal of Energy Research.* 2021; *45* (2):2498-2510.

12. Leng C, Fedoseeva Y V, Zhao Z, et al. Rational-design heteroatom-doped cathode and ion modulation layer modified Zn anode for ultrafast zinc-ion hybrid capacitors with simultaneous high power and energy densities. *J. Power Sources.* 2022; *536* 231484.

13. Yao L, Jiang J, Peng H, et al. Glutinous rice-derived carbon material for high-performance zinc-ion hybrid supercapacitors. *J. Energy Storage.* 2023; *58* 106378.

14. Liu Y, Tan H, Tan Z, Cheng X. Rice husk-derived carbon materials for aqueous Zn-ion hybrid supercapacitors. *Appl. Surf. Sci.* 2023; *608* 155215.

15. Zhang Y, Xie P, Jiang C, Zou Z. Nitrogen and oxygen co-doped carbon micro-foams derived from gelatin as high-performance cathode materials of Zn-ion capacitors. *J. Energy Storage.* 2023; *57*.

16. Zhou G, Li M-C, Liu C, et al. A flexible Zn-ion capacitor based on wood derived porous carbon and polyacrylamide/cellulose nanofiber hydrogel. *Ind. Crops Prod.* 2023; *193* 116216.

17. Yin J, Zhang W, Wang W, Alhebshi N A, Salah N, Alshareef H N. Electrochemical zinc ion capacitors enhanced by redox reactions of porous carbon cathodes *Adv. Energy Mater.* 2020; *10* (37):2001705.

18. Qiu B, Wei X, Zhang W, Lv Y, Meng H, Wei F. Shrimp shell-derived N, O-doped honeycomb-carbon for high-performance supercapacitor. *Diamond Relat. Mater.* 2023; *136* 110041.

19. Li J, Zhang J H, Yu L, et al. Dual-doped carbon hollow nanospheres achieve boosted pseudocapacitive energy storage for aqueous zinc ion hybrid capacitors. *Energy Storage Mater.* 2021; *42* 705-714.

20. Liu P G, Liu W F, Huang Y P, Li P L, Yan J, Liu K Y. Mesoporous hollow carbon spheres boosted, integrated high performance aqueous Zn-Ion energy storage. *Energy Storage Mater.* 2020; *25* 858-865.

21. Shang K, Liu Y, Cai P, Li K, Wen Z. N, P, and S co-doped 3D porous carbon-architectured cathode for high-performance Zn-ion hybrid capacitors. *J. Mater. Chem. A.* 2022; *10* (12):6489-6498.

22. He H, Lian J, Chen C, Xiong Q, Li C C, Zhang M. Enabling multi-chemisorption sites on carbon nanofibers cathodes by an in-situ exfoliation strategy for high-performance Zn–ion hybrid capacitors. *Nano-Micro Lett.* 2022; *14* (1):106-121.

23. Li J, Yu L, Wang W, et al. Sulfur incorporation modulated absorption kinetics and electron transfer behavior for nitrogen rich porous carbon nanotubes endow superior aqueous zinc ion storage capability. *J. Mater. Chem. A.* 2022; *10* (17):9355-9362.

24. Wang D, Pan Z, Lu Z. From starch to porous carbon nanosheets: Promising cathodes for high-performance aqueous Zn-ion hybrid supercapacitors. *Microporous Mesoporous Mater.* 2020; *306* 110445.

25. Wei F, Xu P, Xu C, Han M, Ran S, Lv Y. High-rate performance zinc-ion hybrid capacitors constructed by multi-layered carbon nanosheet cathode. *Ionics.* 2022; *28* (3):1419-1426.
